# UV-B Filter Octylmethoxycinnamate Induces Vasorelaxation by Ca^2+^ Channel Inhibition and Guanylyl Cyclase Activation in Human Umbilical Arteries

**DOI:** 10.3390/ijms20061376

**Published:** 2019-03-19

**Authors:** Margarida Lorigo, Carla Quintaneiro, Manuel C. Lemos, José Martinez-de-Oliveira, Luiza Breitenfeld, Elisa Cairrao

**Affiliations:** 1 CICS-UBI, Health Sciences Research Centre, University of Beira Interior, 6200-506 Covilhã, Portugal; margarida.lorigo@gmail.com (M.L.); mclemos@fcsaude.ubi.pt (M.C.L.); jmo@fcsaude.ubi.pt (J.M.-d.-O.); luiza@fcsaude.ubi.pt (L.B.); 2 Department of Biology & CESAM, University of Aveiro, 3810-193 Aveiro, Portugal; cquintaneiro@ua.pt

**Keywords:** UV-B filter, endocrine disruptor compound, human umbilical artery, vascular smooth muscle cells, relaxation, L-Type VOCC, soluble guanylyl cyclase, organ bath, planar cell surface area

## Abstract

Ultraviolet (UV) filters are chemicals widely used in personal care products (PCPs). Due to their effect as endocrine disruptor compounds (EDCs), the toxicity of UV filters is a current concern for human health. EDC exposure may be correlated to cardiovascular diseases (CVD), but to our knowledge, no studies assessed the UV filters effects as human EDCs at the vascular level. Octylmethoxycinnamate (OMC) is the world’s most widely used UV-B filter, present in more than 90% of PCPs. Due to its demonstrated multiple hormonal activities in animal models, this substance is also suspected to be a human EDC. The purpose of this study was to assess the rapid/short-term effects of OMC on arterial tonus and analyse its mode of action (MOA). Using human umbilical arteries, the endocrine effects of OMC were evaluated in in vitro (cellular and organ) experiments by planar cell surface area (PCSA) and organ bath, respectively. Our data show that OMC induces a rapid/short-term smooth muscle relaxation acting through an endothelium-independent MOA, which seems to be shared with oestrogens, involving an activation of soluble guanylyl cyclase (sGC) that increases the cyclic guanosine monophosphate (cGMP) intracellular levels and an inhibition of L-type voltage-operated Ca^2+^ channels (L-Type VOCC).

## 1. Introduction

Ultraviolet (UV)-B filters are chemical substances widely used in personal care products (PCPs), which can act as endocrine disruptor compounds (EDCs) [1,2]. Due to their capacity to interfere with endogenous hormones [3,4], the toxicity of these EDCs is a current concern for human health, mainly in early stages of development such as in embryos, foetuses, infants and children [5,6,7]. Since the endocrine system controls many maturation processes of organisms (including gestation, infancy and childhood) [6,7], it is crucial to assess the effects of these compounds on human fluids or tissues.

Human umbilical artery (HUA) is a human vascular model to study cardiovascular diseases (CVD) [8], allowing the study of the effect of EDCs, like UV-B filters, on vascular functions of pregnant women and foetuses. HUA is involved in fetoplacental circulation and is an excellent source of vascular smooth muscle cells (SMC) [8,9]. Due to their specific physiological regulation, in vitro studies concerning the intracellular mechanisms modulating HUA contractility are of great importance. These studies may lead to a better understanding of several vascular diseases, mainly hypertension in pregnancy or preeclampsia [8]. Nowadays, many research groups are working to correlate human exposure to EDCs and cardiovascular diseases (CVD) [5,10,11,12]. However, to the best of our knowledge no studies were performed to explore the mode of action (MOA) of UV-B filters as human EDCs and to understand their involvement in vascular diseases.

Octylmethoxycinnamate (OMC) is the world’s most widely used UV-B filter [13]. This substance is suspected to be an EDC [14,15] due to its demonstrated interaction with oestrogen, androgen, progesterone and thyroid receptors [16]. Studies on the toxicity of this UV-B filter were focused primarily on animal models and demonstrated multiple hormonal activities: oestrogenic [14,15,17,18,19,20,21,22,23], antiandrogenic [24,25,26], antiprogestenic [17,25] and antithyroid [19,25,27,28,29]. However, toxicity studies of OMC in humans are scarce.

This issue is of special importance because, as a UV-B filter, OMC can bioaccumulate in the aquatic ecosystems [30,31] and has greater environmental repercussions with potential risks to human health [16,32]. For instance, recently the US state of Hawaii has banned sunscreens containing OMC due the potential risks posed to corals [33]. Moreover, it has been demonstrated that OMC can be transmitted to the zebrafish offspring and produce toxic effects (by parental transfer) [31]—findings that support our hypothesis that toxic effects of OMC might also be observed in humans.

In this context, and due to their widespread presence in a large number of PCPs (more than 90%) [34], in the present work, the UV-B filter octylmethoxycinnamate was selected to assess the rapid/short-term effects on arterial tonus and to discover the underlining mechanisms involved in these effects. To elucidate this, HUA were used in in vitro organ experiments (by organ bath techniques) and the OMC effect was evaluated on the contracted endothelium-denuded HUA rings. The HUA were also used to perform cultures of SMC. The HUASMC cultured were used for in vitro cellular experiments (by planar cell surface area, PCSA), and the OMC effect on the contracted cells was analysed.

## 2. Results

### 2.1. Effects of OMC on Arterial Contractility

The denuded-HUA rings were contracted with two different receptor agonists (serotonin, 5-HT or histamine, His) or by depolarisation with isosmotic KCl (60 mmol/L) solution, to analyse their sensitivity. The maximum contractile effects elicited by 5-HT, His and KCl were 1737 ± 574 mg (*n* = 18), 1054 ± 424 mg (*n* = 21) and 1578 ± 658 mg (*n* = 18), respectively, being 5-HT and KCl significantly different from His (*p* < 0.05, one-way ANOVA with Tukey’s post-hoc test). Then, the OMC effect was examined exposing the contracted arteries to different cumulative concentrations of OMC (0.001–50 μmol/L). All vascular effects observed were reversible after washing with Krebs’ solution.

**Figure 1:**
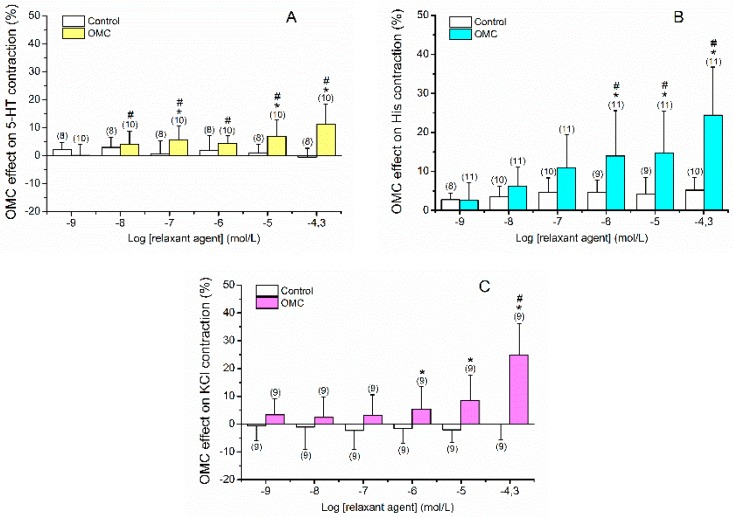
Vasorelaxant effects of octylmethoxycinnamate (OMC, 0.001–50 μmol/L) on endothelium-denuded HUA rings contracted with (**A**) serotonin (5-HT, 1 μmol/L), (**B**) histamine (His, 10 µmol/L) and (**C**) potassium chloride (KCl, 60 mmol/L). Data are expressed as percentage (%) of relaxation on contractile effects. The bars represent the mean values and the lines the standard deviation (S.D.) of the number of artery rings (*n*) indicated above the bars. * Represents statistical differences between OMC and control (*p* < 0.05, Student’s *t*-test) and # represents statistical differences between OMC concentrations (*p* < 0.05, one-way ANOVA followed by Tukey’s post-hoc tests).

OMC induced vasorelaxation of HUA rings precontracted with either serotonin (Figure 1A), histamine (Figure 1B) or KCl (Figure 1C). The OMC effects on 5-HT contractions were significant at concentrations OMC of 0.1, 10 and 50 μmol/L (*p* < 0.05, Student *t*-test), evidencing a nonmonotonic response. All OMC concentrations induced vasorelaxation when compared to the lowest one (0.001 μmol/L) (*p* < 0.05, one-way ANOVA with Tukey’s post-hoc test). However, a monotonic response was observed when His and KCl precontracted arteries were exposed to 1–50 μmol/L of OMC (*p* < 0.05, Student’s *t*-test). For His contractions, the three highest OMC concentrations (1–50 μmol/L) caused a significant vasorelaxation compared with the remaining concentrations (*p* < 0.05, one-way ANOVA with Tukey’s post-hoc test), while in KCl contractions, the highest (50 µmol/L) OMC concentration caused a significantly higher relaxation compared with the other concentrations used (*p* < 0.05, one-way ANOVA with Tukey’s post-hoc test).

As shown in Figure 1, the maximum relaxation induced by OMC in all contractions analysed was observed at the highest tested concentration (50 μmol/L). The relaxations elicited by OMC (50 μmol/L) on 5-HT-, His- or KCl-contracted arteries were 11.31 ± 7.13% (*n* = 10), 24.44 ± 12.31% (*n* = 11) and 24.91  ± 11.36% (*n* = 9), respectively, His and KCl being significantly different from 5-HT (*p* < 0.05, one-way ANOVA with Tukey’s post-hoc test). So, these effects may depend on the contractile agent used. Ethanol (the solvent used to dissolve OMC) did not have significant relaxant effects on contracted arteries at the concentrations used (Figure 1). Concerning the gender of newborns, in all the 24 denuded-HUA rings used for the arterial contractility experiments, nine were from male and 15 were from female foetuses. No gender-specific differences were observed in the OMC effects on 5-HT-, His- or KCl-contracted arteries from males or females (*p* > 0.05, Student’s *t*-test, data not shown).

### 2.2. Effects of L-Type VOCC on OMC-Induced Vasorelaxation 

The denuded-HUA rings were contracted with the same agonists (1 µmol/L 5-HT or 10 µmol/L His) or by depolarisation with isosmotic KCl (60 mmol/L) solution, as previously described. The maximum contractile effects elicited by 5-HT, His and KCl were 1846 ± 158 mg (*n* = 16), 1046 ± 515 mg (*n* = 21) and 1715 ± 530 mg (*n* = 13), respectively, 5-HT and KCl being significantly different from His (*p* < 0.05, Kruskal–Wallis by ranks with Dunn’s post-hoc test). The contracted arteries were exposed to a specific inhibitor of L-Type VOCC (nifedipine, Nif) and the OMC-induced vasorelaxation (OMC; 0.001–50 μmol/L) was examined. Nif (0.1 and 1 μmol/L) was used to analyse the involvement of this type of Ca^2+^ channels in the relaxing effect mediated by OMC. After washing out with Krebs’ solution all observed vascular effects were revered.

**Figure 2:**
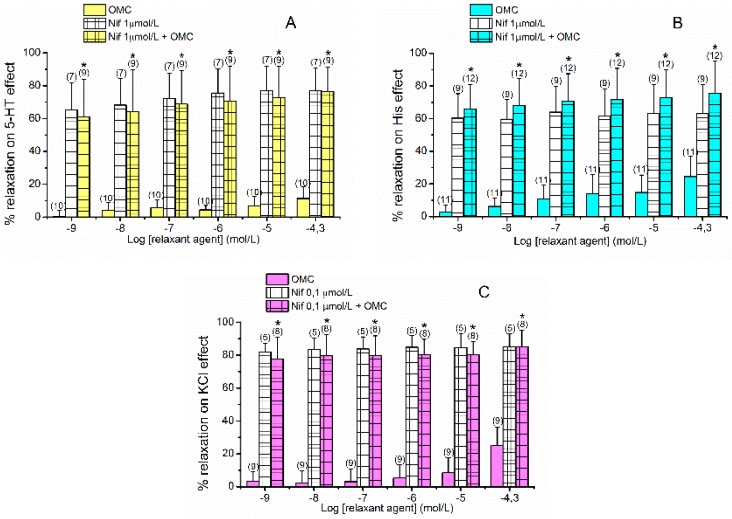
Vasorelaxant effects of octylmethoxycinnamate (OMC, 0.001–50 μmol/L), nifedipine (Nif, 1 μmol/L) and Nif plus OMC on endothelium-denuded HUA rings contracted with (**A**) serotonin (5-HT, 1 µmol/L), (**B**) histamine (His, 10 µmol/L) and (**C**) potassium chloride (KCl, 60 mmol/L). Data are expressed as percentage (%) of relaxation on contractile effects. The bars represent the mean values and the lines the standard deviation (S.D.) of the number of artery rings (*n*) indicated above the bars. * Represent statistical differences between OMC and Nif + OMC (*p* < 0.05) and # represents statistical differences between Nif and Nif + OMC (*p* < 0.05), one-way ANOVA method followed by Tukey’s post-hoc tests or the corresponding nonparametric method the Kruskal–Wallis followed by Dunn’s post-hoc tests.

As shown in the Figure 2, Nif caused vasorelaxation in all contractions analysed. The maximum relaxant effects elicited by Nif on 5-HT-, His- and KCl-contracted arteries were 79.47 ± 13.51% (*n* = 7), 63.99  ± 15.90% (*n* = 9) and 85.31 ±  7.64% (*n* = 5), respectively. The KCl-contracted HUA induced its contraction due to the influx of extracellular Ca^2+^, because of depolarisation and opening of voltage-dependent channels (mainly L-Type VOCC). For this reason, Nif 1 µmol/L (a specific blocker of L-type VOCC) induced a relaxation close to 100% (data not shown), so we used a lower concentration 0.1 µmol/L to better analyse a possible additive effect of the joint use of OMC and Nif. Regardless of the contractile stimuli, the vasorelaxation induced by the joint application of Nif and OMC was significantly different from the individual OMC effect and similar to the individual Nif effect (*p* > 0.05, one-way ANOVA with Tukey’s post-hoc test or Kruskal–Wallis by ranks with Dunn’s post-hoc test). These results suggest that the OMC relaxant effect is mediated by inactivation of L-Type VOCC.

### 2.3. Effects of cGMP on OMC-Induced Vasorelaxation

For the denuded-HUA rings, contraction the same receptor agonists (5-HT; 1 µmol/L or His; 10 µmol/L) or by depolarisation with isosmotic KCl (60 mmol/L) solution were used once more. The maximum contractile effects elicited by 5-HT, His and KCl were 1754 ± 720 mg (*n* = 15), 945 ± 355 mg (*n* = 14) and 1725 ± 330 mg (*n* = 12), respectively, 5-HT and KCl being different from His (*p* < 0.05, Kruskal–Wallis by ranks with Dunn’s post-hoc test). The contracted arteries were exposed to a stimulator of sGC, the sodium nitroprusside (SNP) and the OMC-induced vasorelaxation (OMC; 0.001–50 μmol/L) was examined. SNP (1 and 10 μmol/L) was used to analyse the involvement of this pathway in the relaxing effect mediated by OMC. After washing out with Krebs’ solution all observed vascular effects were reversed.

**Figure 3:**
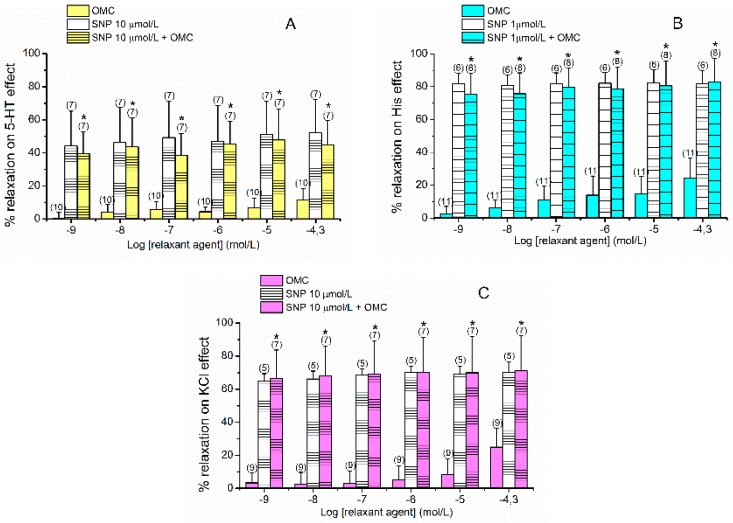
Vasorelaxant effects of octylmethoxycinnamate OMC (0.001–50 μmol/L), sodium nitroprusside (SNP, 10 or 1 μmol/L) and SNP plus OMC on endothelium-denuded HUA rings contracted with (**A**) serotonin (5-HT, 1 µmol/L), (**B**) histamine (His, 10 µmol/L) and (**C**) potassium chloride (KCl, 60 mmol/L). Data are expressed as percentage (%) of relaxation on contractile effects. The bars represent the mean values and the lines the standard deviation (S.D.) of the number of artery rings (*n*) indicated above the bars. * Represent statistical differences between OMC and SNP + OMC (*p* < 0.05) and # represents statistical differences between SNP concentrations and SNP + OMC (*p* < 0.05), one-way ANOVA followed by Tukey’s post-hoc tests or the corresponding nonparametric method the Kruskal–Wallis followed by Dunn’s post-hoc tests).

As shown in Figure 3, SNP caused vasorelaxation in all contractions analysed. The maximum relaxant effects elicited by SNP on 5-HT-, His- and KCl-contracted arteries were 52.05  ±  20.10% (*n* = 7), 82.19 ± 8.04% (*n* = 6) and 70.09 ± 6.57% (*n* = 5), respectively. As for His-contracted HUA, 10 µmol/L of SNP induced a relaxation close to 100% (data not shown), for this reason we used a lower concentration 1 µmol/L to better analyse a possible additive effect of the joint use of OMC and SNP. Regardless of the contractile stimuli, the vasorelaxation induced by the joint application of SNP and OMC was significantly different from the individual OMC effect and similar to the individual SNP effect (*p* > 0.05, one-way ANOVA with Tukey’s post-hoc test or Kruskal–Wallis by ranks with Dunn’s post-hoc test). These results suggest that the OMC relaxant effect is mediated by activation of sGC, with increases of cGMP levels.

### 2.4. Effects of OMC on Cellular Contractility

HUASMC were contracted by two different receptor agonists (5-HT; 1 µmol/L or His; 10 µmol/L). The maximum contractile effects elicited by 5-HT and His were 27.431 ± 4.729 µm^2^ (*n* = 6) and 28.447 ± 7.783 µm^2^ (*n* = 7), respectively. The contracted cells were exposed to a concentration of OMC (50 μmol/L), and the direct effect of OMC on these contractions was examined. The changes in the area of HUASMC were quantified over the time, and in the presence and absence of the different drugs and agents used.

**Figure 4:**
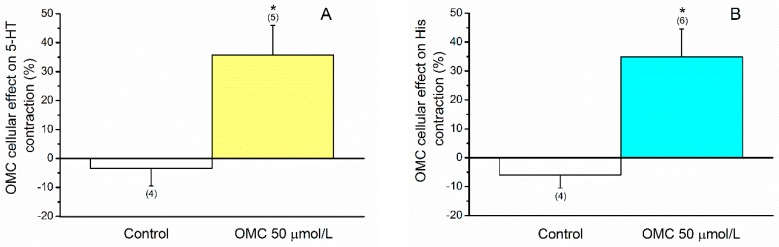
Vasorelaxant effects of octylmethoxycinnamate (OMC, 50 μmol/L) on HUASMC contracted with (**A**) serotonin (5-HT, 1 µmol/L) and (**B**) histamine (His, 10 µmol/L). Data are expressed as percentage (%) of relaxation on area induced by each contractile agent. The bars represent the mean values and the lines the standard deviation (S.D.) of the number (*n*) of human umbilical arteries indicated above the bars. * Represents statistical differences between OMC concentration and control (*p* < 0.05, Student’s *t*-test).

**Figure 5:**
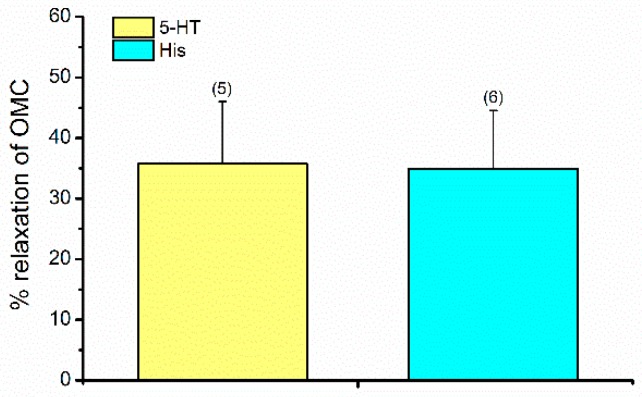
Vasorelaxant effects of octylmethoxycinnamate (OMC, 50 μmol/L) on HUASMC contracted with serotonin (5-HT, 1 µmol/L) and histamine (His, 10 µmol/L). Data are expressed as percentage (%) of relaxation on the area induced by each contractile agent. The bars represent the mean values and the lines the standard deviation (S.D.) of the number (*n*) of human umbilical arteries indicated above the bars. * Represents statistical differences between 5-HT and His (*p* < 0.05, Student’s *t*-test).

After incubation with OMC, a relaxation of the HUASMC was evident (*p* < 0.05, Student’s *t*-test) in cells precontracted either by 5-HT (Figure 4A) or by His (Figure 4B). The relaxing effect elicited by OMC on 5-HT- or His-precontracted cells were 35.767 ± 10.218 µm^2^ (*n* = 5) and 34.898 ± 9.626 µm^2^ (*n* = 6), respectively. Ethanol (the solvent used to dissolve OMC) did not have significant effects on the contracted cells at the concentrations used (Figure 4). As shown in Figure 5, the relaxation data obtained were similar for both contractile agents used (*p* > 0.05, Student’s *t*-test).

## 3. Discussion

Octylmethoxycinnamate is the world’s most widely used UV-B filter [13]. This substance is suspected to be an EDC [14,15] due to its demonstrated interaction with oestrogen, androgen, progesterone and thyroid receptors [16]. However, to date, and to the best of our knowledge, there are no studies performed on human blood vessels. One question remains unanswered: How does OMC act on human arteries contractility? This work aimed to analyse the effect of OMC at the vascular level, and with it we demonstrated for the first time that OMC is a vasodilator of human umbilical arteries.

Using the organ bath technique, firstly, we studied the rapid/short-term effects of OMC on contracted endothelium-denuded HUA. The vascular endothelium was previously removed (because our proposal was to study the effect of OMC at the smooth muscle level) and then cumulative concentrations of OMC (0.001–50 μmol/L) were administered to HUA contracted either with 5-HT, His and KCl (60 mmol/L). As expected, and in accordance with other authors [35,36], our results showed similar maximum contractions induced by 5-HT and KCl (60 mmol/L) in HUA. Nevertheless, other investigators indicated that contractions caused by 5-HT may be higher than those induced by KCl (60 mmol/L) [37]. The tension produced by His was lower than that caused by 5-HT, which is in good agreement with previous studies of Quan et al. (2003) [38] and Cairrao et al. (2008) [36], and by KCl (60 mmol/L). Concerning the maximum OMC-associated effects, as already mentioned, these effects in human arteries are not described in the literature. Results from present work demonstrate for the first time that OMC induces a rapid/short-term and concentration-dependent relaxation of denuded HUA rings contracted with either 5-HT, His or KCl (60 mmol/L). Furthermore, we observed that OMC effects were rapid and reversible, as they disappeared after drug washing. Because the OMC effects were observed in the absence of the endothelium, we can conclude that this vasorelaxant effect is not due to nitric oxide (NO) production, therefore being endothelium-independent. On the other hand, the maximum relaxation induced by OMC in all contractions analysed was observed for the highest concentration tested (50 μmol/L). However, the vasorelaxation in arteries contracted with 5-HT was less pronounced that the effect induced on the arteries contracted with His or KCl (60 mmol/L), where the maximum relaxation achieved was similar. So, these results suggest that effects of OMC were dependent on the contractile agent used. This difference could give some indications as to what MOA might be involved in OMC-induced relaxation. Furthermore, as can be explained based on the different mechanisms involved in the contractile effects of each of these agents, the 5-HT pathway, in the contraction of HUA without endothelium, involves the activation of 5-HT_2A_ receptors (coupled to G_q_ protein) and partial activation of 5-HT_1B_/5-HT_1D_ (coupled to G_i/o_ protein) present in the smooth muscle of this artery. Activation of these receptors leads to muscle contraction [39,40]. In the case of His, contraction is achieved by the activation of H_1_ receptors. This receptor, coupled to G_q_ protein, activates the PLC/IP_3_ signalling cascade, leading to an increase in intracellular Ca^2+^ levels and consequent contraction [40,41,42]. Schneider et al. (2004) also reported the expression of the H_2_ receptor in HUA smooth muscle [43]. This receptor, coupled to G_s_ protein, stimulates adenyl cyclase, leading to an increase in cAMP levels and consequent relaxation. Although the effect of activation of the H_1_ receptor seems to predominate, the effect of His may also be influenced by the activation of the H_2_ receptor, causing less potent contractions. For this reason, we can hypothesise that OMC relaxation in HUA may be due to the action of UV-B filter in the H_1_ or H_2_ receptors and involves Ca^2+^ channels inhibition or K^+^ channel activation. Consistently, the data for 5-HT-contracted HUA seems to indicate that the OMC effect is due to modulation of the 5-HT_2A_ or 5-HT_1B_/5-HT_1D_ receptors and involves Ca^2+^ channels inhibition or K^+^ channels activation. With respect to KCl, the vascular contraction induced by this agent is mainly due to the influx of extracellular Ca^2+^, because of depolarisation and opening of voltage-dependent channels (mainly L-Type VOCC) [37]. These channels open and increase intracellular Ca^2+^ concentration [Ca^2+^]_I_ resulting in muscle contraction [44]. Thus, taking into account that [Ca^2+^]_I_ is a key to HUA vascular smooth muscle contraction/relaxation [8], our results seem to be consistent with the inhibition of L-Type VOCC as the main pathway involved in the OMC vasorelaxation.

Consequently, to check this pathway, the next step was to analyse the MOA of OMC, exploring the role of this type Ca^2+^ channels on OMC-induced vasorelaxation. For this, contracted endothelium-denuded HUA rings by different stimuli were exposed to a specific inhibitor of L-Type VOCC (nifedipine, Nif) and the OMC-induced vasorelaxation was examined. As expected, and in accordance with other authors (Saldanha et al., 2013) [45], our results showed that Nif induces relaxation on HUA precontracted with 5-HT, His or KCl (60 mmol/L). However, the fact that Nif 1 µM induces total relaxation of KCl-contracted HUA (data not shown) led us to use a lower concentration of Nif (0.1 µmol/L) to better analyse a possible additive effect of the joint use of OMC and Nif. Moreover, and despite being not statistically different, it was visible that the maximum relaxant effects elicited by Nif 0.1 μmol/L on KCl-contracted arteries remained slightly larger than the effects elicited by Nif 1 μmol/L on contracted arteries with 5-HT and His. Therefore, these results confirm that the KCl-contraction is due to the opening of the L-Type VOCC. Concerning the OMC-associated effects, independently of the contractile stimuli, it was evident that the vasorelaxation induced by the joint application of Nif and OMC was significantly different from the individual OMC-induced effect and was similar to the individual Nif-induced effect. These data support the idea that OMC can have the same MOA as Nif, or act through an interconnected pathway that involves inactivation of voltage-dependent Ca^2+^ channels, L-Type VOCC.

Several studies have reported an oestrogenic activity for OMC [14,15,17,18,19,20,21,22,23]. Currently, it is clear that oestrogens cause rapid vasodilation at the arterial level [46,47,48,49,50] and in HUA an oestrogen-mediated vasorelaxation has been previously reported [51,52]. Specifically, it has been demonstrated that one of the privileged pathways for oestrogen-mediated relaxation occurs through a rapid and endothelium-independent mechanism [47,49,53], which is in good agreement with the data we obtained. Concerning L-Type VOCC inhibition, it has also been shown that inhibition of these Ca^2+^ channels is associated with oestrogen-mediated vasodilation [49,50,54,55]. For instance, Okabe et al. (1999) demonstrated that 17β-oestradiol inhibits Ca^2+^ channels in SMC from pregnant rat myometrium [50], while Zhang et al. (2002) also reported the same inhibitory effect of oestradiol on L-Type VOCC in A7r5 cells [56]. Moreover, other authors demonstrated that 17β-oestradiol induces rapid and endothelium-independent relaxation by inhibiting L-Type VOCC in vascular SMC [57] and in rat aortic smooth muscle [49]. As expected, and in accordance with these authors our findings show that OMC induces vasorelaxation by inhibition of L-type VOCC in human umbilical arteries. Hence, the relaxing effect of OMC is endothelium-independent, and its MOA may involve the inactivation of Ca^2+^ channels, L-Type VOCC. Taken together, these data have led us to create the following question. Is the vascular MOA of OMC to induce vasorelaxation in HUA similar to that of oestrogens?

To answer this question, the next step was to analyse the MOA of OMC, exploring the role of cyclic nucleotide (soluble guanyl ciclase, sGC) on OMC-induced vasorelaxation. This was chosen because the activation of sGC (which increases the cGMP intracellular levels) has been proven to be one of the main pathways by which oestrogens induce vascular relaxation [40,58]. Several authors have demonstrated increases in cGMP levels associated with the vasodilator effects of 17β-oestradiol in human coronary artery [59] and rat aortic artery [60]. So, to analyse this pathway, contracted endothelium-denuded HUA rings by different stimuli were exposed to a stimulator of sGC (sodium nitroprusside, SNP) and the OMC-induced vasorelaxation was examined. Results showed that SNP induces relaxation on HUA precontracted either with 5-HT or His or KCl (60 mmol/L), as demonstrated in previous HUA studies [40,58]. The SNP-induced relaxation on His-contracted arteries was larger than on 5-HT contracted arteries, which is in accordance with previous studies of Santos-Silva et al. (2008) [40]. Concerning the OMC-associated effects, independently of the contractile stimuli, the vasorelaxation induced by the joint application of SNP and OMC was significantly different from the individual OMC-induced effect and was similar to the individual SNP-induced effect. These data suggest that either SNP or OMC can have the same MOA, which involves sGC, since the effect of SNP plus OMC does not increase. Thus, these results support our hypothesis that the MOA of OMC can be shared with that of oestrogens, also involving activation of sGC with increases in intracellular cGMP levels.

To confirm the effects of OMC on human vasculature, the cell contractility of HUASMC was measured by PCSA technique. Cultures of HUASMC were obtained through explants of the umbilical artery, as described by Martin et al. (2007) [61] and Cairrao et al. (2009) [9]. The established protocol allowed us to successfully obtain HUASMC cultures without contamination by endothelial cells and/or fibroblasts, one of the great limitations in the establishment of SMC cultures. The HUASMC were precontracted by two different receptor agonists (5-HT; 1 µmol/L or His; 10 µmol/L), since it was impossible to perform the depolarisation with isosmotic KCl (60 mmol/L) solution. Then, the contracted cells were exposed to a concentration of OMC (50 μmol/L) and the direct effect of OMC on these contractions was examined. In the organ bath technique, we observed that OMC induces a relaxing effect on HUA. Our PCSA data also showed for the first time a relaxing effect induced by OMC in vascular SMC, which was similar for both contractile agents used. So, these findings support and confirm the observed vasorelaxant effects in HUA rings.

**Figure 6:**
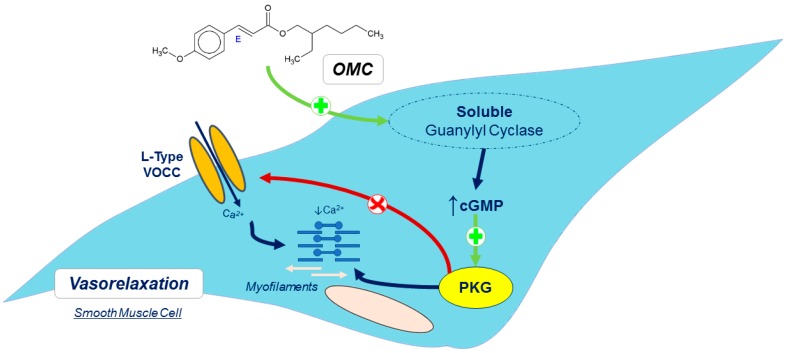
Schematic representation of proposed rapid/short-term MOA of OMC in vascular smooth muscle. OMC crosses the plasma membrane and activates sGC. sGC activation leads to an increase in the level of cGMP that activates PKG. This kinase induces L-Type VOCC inactivation. OMC through the L-Type VOCC inactivation and PKG activation will reduce [Ca^2+^]_i_ and myofilament sensitisation to Ca^2+^, leads to vasorelaxation of the smooth muscle cells. 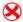/Red arrows—inhibition; 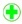/Green arrows—Stimulation; Ca^2+^—calcium; cGMP—cyclic guanosine monophosphate; L-Type VOCC—L-type voltage-operated Ca^2+^ channels; OMC–octylmethoxycinnamate; PKG—protein kinase G.

Taken together, our data show that OMC induces rapid/short-term smooth muscle relaxation acting through an endothelium-independent MOA that seems to be shared with oestrogens, involving an activation of sGC with increases in the cGMP intracellular levels and an inactivation of L-Type VOCC. Our data are consistent with those reported in the literature, which led us to propose a model for vascular MOA of OMC which is illustrated in Figure 6.

## 4. Materials and Methods

Experimental studies were performed in the CICS-UBI laboratories (Health Sciences Research Centre, University of Beira Interior, Covilhã, Portugal). The laboratories have all the necessary equipment to develop the methodologies described below. Centro Hospitalar Universitário da Cova da Beira E.P.E. (CHUCB, Covilhã, Portugal) is one institution with an established collaboration with UBI.

### 4.1. Sample Collection

Umbilical cord (UC) samples were obtained from normal full-term pregnancies after vaginal delivery. All donor mothers were healthy and were under no medication, other than folic acid during the first 21 weeks of gestation or iron supplementation throughout the gestational period. All subjects gave their informed consent for inclusion before they participated in the study. Samples were collected after informed consent. The experiments followed a protocol approved by the Ethics Committee to health of Centro Hospitalar Universitário da Cova da Beira E.P.E. (No.33/2018, 18 July 2018). Samples were resected from the proximal half of the UC (20 cm) and collected within 10 to 20 min after delivery. The UC collected were stored at 4 °C for 4–24 h in sterile physiological saline solution (PSS). Composition of PSS solution: NaCl 110 mmol/L; CaCl_2_ 0.15 mmol/L; KCl 5 mmol/L; MgCl_2_ 2 mmol/L; HEPES 10 mmol/L; NaHCO_3_ 10 mmol/L; KH_2_PO_4_ 0.5 mmol/L; NaH_2_PO_4_ 0.5 mmol/L; Glucose 10 mmol/L; and EDTA 0.49 mmol/L. To avoid contamination, antibiotics (penicillin, 5 U/mL, streptomycin, 5 μg/mL and amphotericin B, 12.5 ng/mL) were added to the PSS solution. In addition, to avoid tissue degradation, antiproteases (leupeptin, 0.45 mg/L; benzamidine, 26 mg/L; and trypsin inhibitor, 10 mg/L) were added to the same solution.

### 4.2. Arterial Contractility Experiments

The dissection and treatment of arteries were performed as described by Cairrao et al. (2008) [36]. Briefly, UC pieces were placed on glass Petri dishes containing PSS solution and the arteries were isolated by removal of the surrounding connective tissue (Wharton’s jelly). HUA isolated were cut into small rings (size 3–5 mm) and the vascular endothelium was mechanically removed with a cotton bud introduced through the arterial lumen. Then, these rings were used to perform arterial contractility experiments.

The HUA rings were placed in organ bath chambers LE01.004 (Letica, Madrid, Spain) containing Krebs-bicarbonate solution (20 mL) at a temperature of 37 °C. The composition of Krebs modified solution was NaCl 119 mmol/L, KCl 5.0 mmol/L, NaHCO_3_ 25 mmol/L, KH_2_PO_4_ 1.2 mmol/L, CaCl_2_ 0.5 mmol/L, MgSO_4_ 1.2 mmol/L, EDTA 0.03 mmol/L and glucose 11 mmol/L (pH 7.4). The rings were suspended between two parallel stainless-steel wires and the tension was measured in millinewton (mN) using isometric transducers TRI201 (Panlab SA, Madrid, Spain) connected to an ML118/D Quad Bridge amplifier (AD Instruments, Oxford, UK), an interface Power Lab/4SP ML750 (ADInstruments) and a computerised system with Chart 5 Power Lab software (ADInstruments). The artery rings were continuously aerated with carbogen (95% O_2_ and 5% CO_2_), because this gas mixture allowed the CO_2_ pressure and pH value in the organ bath to be similar to the values in human plasma.

The artery rings were placed under a preresting tension (20–25 mN) and were subjected to an equilibration period for 60 min. During this period, the organ bath solution was changed every 15 min. The viability of rings was tested by precontracting them with a supramaximal concentration of 5-HT (1 μmol/L) and the rings in which a maximum contraction <10 mN were not used in the study [36]. The vascular effect of OMC on 5-HT (1 μmol/L), His (10 μmol/L) or KCl (60 mmol/L) contractions was evaluated: cumulative OMC concentrations (0.001, 0.01, 0.1, 1, 10 and 50 μmol/L) were added. The OMC concentrations were chosen according to Schlumpf et al. 2001 [14].

To determine the involvement of Ca^2+^ channels in OMC-induced vasorelaxation, nifedipine (Nif, a specific inhibitor of L-Type VOCC and the contractile agent KCl (60 mmol/L) (that induce depolarisation of SMC membrane by opening L-Type VOCC) was used. After a stable contraction with the different contractile agents, the rings were incubated with Nif (0.1 or 1 μmol/L) and the vasorelaxation induced by OMC (0.001–50 μmol/L) was analysed.

The involvement of cyclic nucleotides in OMC-induced vasorelaxation was also investigated. For this, sodium nitroprusside (SNP, a soluble guanylyl cyclase (sGC) stimulator) was used. After a stable contraction with the different contractile agents, the rings were incubated with SNP (1 or 10 μmol/L) and the vasorelaxation induced by OMC (0.001–50 μmol/L) was analysed.

Control experiments with ethanol were always performed. Each experiment was conducted in a several HUA rings from at least three different arteries. Because SNP, Nif and OMC are photodegradable agents, all procedures were carried out in absence of light.

### 4.3. Cell Dissociation and Culture

Cultures of HUASMC were obtained through explants of the umbilical artery, as described by Martin et al. (2007) [61] and Cairrao et al. (2009) [9]. All procedures were performed inside a laminar flow chamber after aseptic procedures and using sterile materials, solutions and instruments. Briefly, UC pieces were placed in glass Petri dishes containing PSS solution and antibiotics. HUA were isolated from the UC pieces by removal of the surrounding connective tissue. The smooth muscle layers from the tunica media were extracted and to avoid endothelial cell contamination, the tunica intima was mechanically removed by gentle rubbing with a cotton bud. In this process, the circular media layer that is in contact with the adventitia was rejected to guarantee the total removal of fibroblasts [8]. Pieces of longitudinal media layer were washed 4 times with PSS, for 5 min each wash. This mechanical dissociation with a Pasteur pipette allows the removal of any cells that could still be present or tissue debris. After that, pieces were distributed in culture dishes (coated with collagen, 20 μg/cm^2^) and the excess of PSS surrounding the tissue pieces was removed. Dishes were incubated (10 to 15 min) at 37 °C in an atmosphere of 95% O_2_ and 5% CO_2_ to dry and, thus, facilitate the tissue pieces adsorption on the surface. Then, culture medium was added. The composition of the cell culture medium was DMEM-F12 containing bovine serum albumin (BSA, 0.5%), heat-inactivated foetal bovine serum (FBS, 5%), epidermal growth factor (EGF, 5 μg/mL), fibroblast growth factor (FGF, 0.5 ng/mL), heparin (2 μg/mL), insulin (5 μg/mL) and a mixture of antibiotics: penicillin (5 U/mL), streptomycin (5 μg/mL) and amphotericin B (12.5 ng/mL). SMC migrated from the tissue to the culture dish surface, where they grew at 37 °C in an atmosphere of 95% O_2_ and 5% CO_2_. The culture medium was changed every 2–3 days to obtain confluent cultures (20–30 days). At this point (when HUASMC spread out from the explants covering ~90–95% of the dish area), cells were trypsinised, the cell culture medium was removed, the cells were washed with phosphate-buffered saline (PBS) and commercial trypsin-EDTA solution (0.025%) was added to detach the cells from the dish surface. After trypsinisation, the cells were transferred to cell culture flasks and cultured as above. Subcultures of HUASMC were performed until the fourth passage. Cells from the different passages were used to perform PCSA experiments (see below).

### 4.4. Cellular Contractility Experiments

The Planar cell surface area technique was carried out as described by our group [62], working with vascular SMC. The PCSA is a useful technique allowing the study of changes in the cell surface area through the image acquisition of SMC. The recorded images allow us to analyse a decrease or an increase in cell areas that correspond to a contraction or a relaxation, respectively [62].

The HUASMC were grown in 6-well culture plates with culture medium until confluence. At this point, cell culture medium was removed. The HUASMC were placed in culture medium without FBS (FBS-free culture medium) and incubated (24 h) at 37 °C in an atmosphere of 95% O_2_ and 5% CO_2_, once these are the required conditions for SMC to express the contractile phenotype necessary for the study [8,9]. The composition of the FBS-free culture medium was DMEM-F12 containing bovine serum albumin (BSA, 0.5%). After that, cells were trypsinised and plated in specific Petri dishes (coated with collagen, 5 mg/cm^2^) wrapped in silver and incubated (2 h) as above. After this incubation period, the cells were washed 4 times with PBS. The whole procedure was performed in the absence of light due to photodegradation of OMC.

Direct observation of cells was carried out at room temperature under phase contrast with an inverted fluorescence microscope (Zeiss Axio Observer Z1, Jena, Germany). This microscope is equipped with an incubation system that controls temperature (maintaining the cellular viability) and a high-speed monochrome digital camera Axio Cam Hsm (Zeiss) to take photographs of cells. The HUASMC were incubated under different experimental conditions: firstly, 5-HT (1 μmol/L) or His (10 μmol/L) was added and waited until the cell contraction reached its maximal response. Then, the vascular effect of OMC on these contractions was evaluated for 50 μmol/L of OMC, which is the concentration where maximum relaxation was achieved according to the organ bath data. Control experiments with ethanol were always performed. Serial photographs were taken along each experiment, specifically before and after all experimental additions. The PCSA was determined by computerised image analysis using the Axion vision 4.8 software (Zeiss). Actual area measurement was calculated using the supplementary “Automatic Measurement program” (Zeiss). Four to eight cells per photograph were chosen for analysis, and a suitable sharp margin for its planimetric analysis was always considered. In every experiment, a set of cells from only single culture and passage were used. The same experimental protocol was repeated in cells from different passages and different cultures.

### 4.5. Drugs and Chemicals

All the drugs and chemicals were purchased from Sigma-Aldrich Química (Sintra, Portugal). To create stock solutions, the different drugs were diluted to desired concentration with distilled water or absolute ethanol. Octylmethoxycinnamate (OMC) and nifedipine (Nif) were dissolved in ethanol. All the solutions were stored at −20 °C. Final solutions of OMC and ethanol control were obtained by dilution with Krebs solution or FBS-free culture medium. These appropriate dilutions were carried out according to the experiment and were prepared daily. The final concentration of ethanol never exceeded 0.05% in all experiments.

### 4.6. Statistical Analysis

For statistical analysis of arterial contractility experiments, the isometric tension measurements were expressed in millinewton (mN) of force elicited by the artery in the presence of vasoconstrictor drugs (5-HT, His or KCl 60 mmol/L). The relaxant responses induced by OMC were expressed as a % of reduction of the maximal contraction induced by each contractile agent. All results were expressed as mean ± standard deviation (S.D.) of the number (n) of rings used.

Regarding statistical analysis of cellular contractility experiments, the actual area measurements were expressed in micrometres^2^ (µm^2^) of area achieved by the cell in the presence of vasoconstrictor drugs (5-HT or His). The relaxant responses induced by OMC were expressed as a % of reduction of the maximal area induced by each contractile agent. Results were expressed as mean ± standard deviation (S.D.) of the number (n) of the human umbilical arteries used to obtain the cells.

The software SigmaStat Statistical Analysis System version 3.5 (2006) was used to perform statistical treatment of data, and the graphic design was achieved with Software Origin 8.5.1. Presence of a normal distribution was elucidated using Kolmogorov–Smirnov test. A Student’s *t*-test was used to analyse the statistical significance between two groups. In comparison among multiple groups, one-way ANOVA method followed by Tukey’s post-hoc tests or the corresponding nonparametric method (Kruskal–Wallis) followed by Dunn’s post-hoc tests were used to determine significant differences between the means. For all tests, a *p*-value less than 0.05 was considered statistically significant.

## 5. Conclusions

In conclusion, this work represents a whole new and promising research field that remains almost entirely unexplored. Further studies are needed to increase the knowledge about the effects of OMC at the vascular level. In addition to the human studies, it is important to study its effects on other species and arteries to uncover the cardiovascular toxicity of this UV-B filter. Given its wide presence in the environment and its potentially adverse effects on human health, studying human exposure to OMC may lead to a better understanding of the role of OMC in cardiovascular diseases and the identification of molecular pathways that can be targeted for the prevention and treatment of these diseases.

## Abbreviations

5-HT	Serotonin
cAMP	Cyclic Adenosine Monophosphate
CDV	Cardiovascular Diseases
cGMP	cyclic Guanosine Monophosphate
CHUCB	“Centro Hospitalar Universitário da Cova da Beira E.P.E.”
CICS-UBI	Health Sciences Research Centre, University of Beira Interior
EDCs	Endocrine Disruptor Compounds
His	Histamine
HUA	Human Umbilical Artery
HUASMC	Human Umbilical Artery Smooth Muscle Cells
KCl	Potassium Chloride
L-Type VOCC	L-Type voltage-operated Ca^2+^ channels
MOA	Mode of Action
Nif	Nifedipine
OMC	Octylmethoxycinnamate
PCPs	Personal Care Products
PCSA	Planar Cell Surface Area
PKG	Protein Kinase G
PSS	Sterile Physiological Saline Solution
sGC	soluble Guanylyl Cyclase
SMC	Smooth Muscle Cells
SNP	Sodium Nitroprusside
UC	Umbilical Cord
US	United State
UV	Ultraviolet
